# Items

**Published:** 1886-04

**Authors:** 


### ITEMS.

ANOTHER HIGH-GRADE MEDICAL SCHOOL IN THE NORTHWEST.—On February 3rd, 1886, the new buildings of the ST. PAUL MEDICAL SCHOOL were formally dedicated to the cause of higher medical education.

The St. Paul Medical School was organized in 1871 as a preparatory school, and reorganized on the same basis in 1876. It was organized as a regular medical college with a GRADED COURSE OF FOUR YEARS in 1878. In 1880 the school was transferred to Minneapolis, but in 1885 was brought back to St. Paul.

It is now organized on a basis of a GRADED COURSE OF INSTRUCTION, extending through THREE YEARS.

THE NEUROLOGICAL REVIEW.—It is announced that the first number of *The Neurological Review* will be issued between the 15th of this month and the 1st of May succeeding.

Thereafter the *Review* will appear monthly, under the editorial direction of Professor J. S. Jewell, of Chicago, assisted by Dr. H. M. Bannister, of Kankakee. Its contents will be arranged according to the following plan:

I. Original and Selected Articles.

II. Editorial Department.

III. Review Department.

The success of the *Journal of Nervous and Mental Diseases*,—published until within a few years in Chicago, under the same editorial management,—in collecting, digesting, and discussing neurological science may be accepted as an earnest of what may be expected of the new journal.

The *Medical Analectic*, a monthly periscopic summary of the progress of medical science, published by G. P. Putnam's Sons, will hereafter be edited by R. W. Amidon, A.M., M.D., New York.

INSANITY FROM CATARACT OPERATIONS. — Landesberg (*Medical and Surgical Reporter*, volume LIII.) has had three cases in which mental confusion, violence, visual and auditory hallucinations resulted after cataract operations. That the operation had an influence in producing the mental phenomena is shown by the twelve similar cases reported by Schnabel (Bericht. der Verein für Medic. Wissens. Insbruck Jahrg. XIII). In 186 cataract operations performed by Schnabel, he observed but these twelve cases, all of whom were between 66 and 80 years old; there were ten men and two women. That the operation caused the insanity is shown by the fact that when, in cases of double cataract, the mental symptoms resulting from the operation on one eye had subsided, they were immediately reproduced on the operation being repeated. Sichel (*L'Union Médicale*, No. 1, 1869) was the first to notice this insanity, and it has been suggested (*Journal of Nervous and Mental Diseases*, volume XI) that the approach of senility predisposed to it. As in all insanity of the confusional hallucinated type, the patient rapidly recovers.

